# Insulin-like and IGF-like peptides in the silkmoth *Bombyx mori*: discovery, structure, secretion, and function

**DOI:** 10.3389/fphys.2013.00217

**Published:** 2013-08-16

**Authors:** Akira Mizoguchi, Naoki Okamoto

**Affiliations:** ^1^Division of Biological Science, Graduate School of Science, Nagoya UniversityNagoya, Japan; ^2^Laboratory for Growth Control Signaling, RIKEN Center for Developmental BiologyKobe, Japan

**Keywords:** insulin-like peptide, IGF-like peptide, *Bombyx mori*, bombyxin, BIGFLP, dilp6, insect, growth

## Abstract

A quarter of a century has passed since bombyxin, the first insulin-like peptide identified in insects, was discovered in the silkmoth *Bombyx mori*. During these years, bombyxin has been studied for its structure, genes, distribution, hemolymph titers, secretion control, as well as physiological functions, thereby stimulating a wide range of studies on insulin-like peptides in other insects. Moreover, recent studies have identified a new class of insulin family peptides, IGF-like peptides, in *B. mori* and *Drosophila melanogaster*, broadening the base of the research area of the insulin-related peptides in insects. In this review, we describe the achievements of the studies on insulin-like and IGF-like peptides mainly in *B. mori* with short histories of their discovery. Our emphasis is that bombyxins, secreted by the brain neurosecretory cells, regulate nutrient-dependent growth and metabolism, whereas the IGF-like peptides, secreted by the fat body and other peripheral tissues, regulate stage-dependent growth of tissues.

## Introduction

### The aim of this review

Since 1984, when bombyxin was identified as the first insulin-like peptide in invertebrates (Nagasawa et al., 1984b), this brain neurosecretory hormone of the silkmoth *Bombyx mori* has been extensively characterized for its chemical nature, gene structure, distribution, secretion control, as well as physiological functions. In addition, another class of insulin-related peptides has recently been discovered again in *B. mori* and characterized (Okamoto et al., 2009a). The former is similar to vertebrate insulin in its two-chain structure and physiological functions, while the latter to vertebrate insulin-like growth factors (IGFs) in its single-chain structure, sites of production and physiological roles. In this article, we will provide an overview of the accumulated knowledge on these two classes of *Bombyx* insulin-related peptides and discuss the physiological significance of the presence of these hormones in insects. Recently, physiological functions and action mechanisms of insect insulin/IGF-like peptides have been actively studied in other insects, especially in the fruit fly *Drosophila melanogaster*, by using genetic approaches. In this review, however, we will concentrate on the achievements with *B. mori*, because comprehensive reviews exist for *Drosophila* and other insects (Garofalo, 2002; Oldham and Hafen, 2003; Tatar et al., 2003; Géminard et al., 2006; Wu and Brown, 2006; Broughton and Partridge, 2009; Grewal, 2009; Teleman, 2010; Grönke and Partridge, 2010; Antonova et al., 2012; Nässel, 2012; Hyun, 2013).

### Historical background

Insulin is best known for its hypoglycemic action (Newsholme et al., 1992; Saltiel and Kahn, 2001). Even before the discovery of bombyxin, the existence of hypoglycemic hormones had been demonstrated in the honeybee *Apis mellifera* (Kramer et al., 1977, 1982; Bounias et al., 1986), the blowfly *Calliphora vomitoria* (Duve et al., 1979), the tobacco hornworm *Manduca sexta* (Tager et al., 1976; Kramer et al., 1982), the cockroach *Periplaneta americana* (Barrett and Loughton, 1987) and others (for a review, see Kramer, 1985). These hypoglycemic hormones were presumed to be insulin-related peptides, because insulin-immunoreactive substances had also been detected in many insects including above-mentioned species as well as the locust *Locusta migratoria* and the silkmoth *B. mori* by radioimmunoassay (RIA) (Ishay et al., 1976; Tager et al., 1976; Kramer et al., 1977; Duve et al., 1979; Orchard and Loughton, 1980; Kramer, 1985) and/or immunocytochemistry (Duve and Thorpe, 1979; Yui et al., 1980). These insulin-like peptides in most insects were localized in the neurosecretory cells of the brain and its neurohemal organs, corpora cardiaca (CC) and/or corpora allata (CA), and were therefore recognized as brain neurosecretory hormones. The insulin-immunoreactive material was purified and chemically characterized in *M. sexta* (Kramer et al., 1982) and *C. vomitoria* (Thorpe and Duve, 1984). Although the amino acid sequence was not determined, their molecular size and amino acid composition were similar to those of vertebrate insulins.

## *Bombyx* insulin-like peptide (bombyxin)

### Discovery of bombyxin

Bombyxin was initially called 4K-prothoracicotropic hormone (4K-PTTH), because it was purified as the brain neurosecretory hormone with MW of 4400 that stimulates the prothoracic glands (PGs) to release ecdysone (Nagasawa et al., 1984b, 1986). In this purification study, the adult heads of *B. mori* were used as the starting material for purification and the debrained dormant pupae of the heterologous moth *Samia cynthia ricini* were used for the bioassay of the hormone, because PTTH was believed to be species-nonspecific between these two species and because *Samia* debrained pupae provided a more stable assay system than *Bombyx* debrained pupae (see Ishizaki, 2004 for more detailed history of bombyxin and PTTH purifications). In fact, crude extracts of *B. mori* brain were able to provoke adult development when injected into debrained dormant pupae of both *B. mori* and *S. cynthia ricini*. At the final stage of purification, however, the purified 4K-PTTH was found to be ineffective on the PGs of *B. mori* from which it was derived (Ishizaki et al., 1983), indicating that this peptide is not the true PTTH of *B. mori*. Nevertheless, the name 4K-PTTH was used for years thereafter, because it was actually highly active on the *Samia* PGs both *in vivo* and *in vitro* (Nagasawa et al., 1984a). This peptide was purified to homogeneity and its partial sequence determined in 1984 (Nagasawa et al., 1984a,b). Surprisingly, the N-terminal sequence of 4K-PTTH was structurally homologous to vertebrate insulin and IGF (see section Primary Structure). This was the first insulin-related peptide identified in invertebrates. Thus, this peptide was finally renamed bombyxin for *Bombyx* insulin (Mizoguchi et al., 1987).

### Primary structure

4K-PTTH was purified to homogeneity through 15 steps of purification from 678,000 *Bombyx* adult heads, yielding three peaks on HPLC. Each peak contained 36–50 μ g of peptide, and was sequenced separately. The N-terminal 19 amino acid sequences of these peptides, named 4K-PTTH-I, II, III, were similar to each other and even to the corresponding portions of human insulin and IGF-I (Nagasawa et al., 1984b). The complete amino acid sequence was determined later for 4K-PTTH-II (Nagasawa et al., 1986; for minor revision, see Nagasawa et al., 1988). It consisted of two non-identical peptide chains (A and B chains), like vertebrate insulin. The A and B chains consisted of 20 and 28 amino acid residues, respectively. 4K-PTTH-II showed high sequence identity (~40%) with human insulin, and the positions of seven cysteine residues were completely identical with those in other insulin family peptides.

### Higher structure

The location of disulfide bridges in the molecule was determined through thermolysin digestion of 4K-PTTH-II (hereafter bombyxin-II) and subsequent chemical analyses (Nagasawa et al., 1988). Three disulfide bonds were linked in the same positions as in insulin.

A three-dimensional model of bombyxin-II was constructed using interactive computer graphics and energy minimization techniques (Jhoti et al., 1987), confirming the insulin-like structure of bombyxin-II (called PTTH-II in the paper). This model predicts that bombyxin-II is unlikely to form either dimers or hexamers, characteristic of human insulin and that a hydrophobic surface region of the peptide may be important in binding with other proteins.

In addition to bombyxin-I, -II, -III, two more bombyxin species have been isolated from *Bombyx* heads. The primary structure of bombyxin-IV was determined for both chains (Maruyama et al., 1988), and that of bombyxin-V for the B-chain (Jhoti et al., 1987). Bombyxin-IV had 62.5% sequence identity with bombyxin-II.

Bombyxin-II and -IV were chemically synthesized by solid phase peptide synthesis of the A- and B-chains followed by air oxidation (Nagasawa et al., 1988) or semicontrolled pairing of cysteine residues (Maruyama et al., 1990) in a yield of 4 and 8%, respectively. The synthetic bombyxin-II and -IV had nearly the same specific activity as the natural ones.

By using the synthetic bombyxin-II, its three-dimensional solution structure was determined by NMR measurements (Nagata et al., 1995a). Although the overall main-chain structure of bombyxin-II was similar to that of insulin, significant conformational and functional differences in their B-chain C-terminal portions were found. This part of bombyxin-II adopts an extension of the B-chain central helix like that of relaxin and is not required for bombyxin activity, contrasting with the corresponding part of insulin, which adopts a sharp turn and a β -strand and is essential for insulin activity. A further study employing chimeric molecules of bombyxin-II and human insulin suggested that the surface patch formed by the central part of the bombyxin-II B-chain is of critical importance for recognition of the bombyxin receptor (Nagata et al., 1995b).

### Bombyxin genes

Although the above purification study revealed an abundance of molecular forms of bombyxin, the subsequent molecular cloning studies have identified more genes encoding bombyxins. Thirty-two bombyxin-encoding genes have been cloned (Iwami et al., 1989, 1990; Kawakami et al., 1989; Kondo et al., 1996; Tsuzuki et al., 1997; Yoshida et al., 1997, 1998). In addition, some more bombyxin genes have been identified by the BLAST searches on the *Bombyx* genome (Aslam et al., 2011). The cloned genes are classified into 7 families, A–G, according to their sequence similarity. The families A, B, and C consist of 10, 12, and 6 genes, respectively, and each of the families D–G contains a single gene (Iwami, 2000). Among these genes, A6 and/or A7 encode bombyxin-II, and E1 encode bombyxin-IV. However, the genes coding for bombyxin-I, III, and V have not been identified. Most bombyxin genes encode polypeptides similar to preproinsulin, which consists of the signal peptide, B-chain, C-peptide, and A-chain, in this order, from the N-terminus, although several other genes are presumably pseudogenes (Kondo et al., 1996). Novel bombyxin genes recently identified in the *Bombyx* genome are classified into five different families, V–Z (Aslam et al., 2011). Each family contains a single gene except for family V, which includes two closely related genes. These new bombyxin genes also code for preproinsulin-like structure, similar to the family A–G genes. The similarities in the amino acid sequences of preprobombyxins between families are about 50% (Iwami, 2000). The sequence similarities are high in the A- and B-chains (domains) but low in the C-peptide (domain).

All six cysteines and some hydrophobic residues in the A- and B-domains responsible for the formation of a hydrophobic core are completely conserved in all probombyxins and insulin. The C-domains of all probombyxins are flanked by dibasic (monobasic in some cases) sites, suggesting that the C-domain is removed to generate mature bombyxins in the same way as insulin maturation. However, this may be the case only when the bombyxin genes are expressed in specific tissues such as the brain, from which bombyxin-II and -IV were purified. These two bombyxins have been confirmed to consist of A- and B-chains like insulin, as described above. In 2009, however, a novel insulin family peptide containing the C-domain was purified from *Bombyx* pupal hemolymph [(Okamoto et al., 2009a); see section *Bombyx* IGF-Like Peptide (BIGFLP) for details]. This peptide, named *Bombyx* IGF-like peptide (BIGFLP), is the product of the bombyxin Y1 gene (Aslam et al., 2011), and its C-domain is also flanked by dibasic sites. However, this peptide is secreted with the C-domain intact. Its main production site is the fat body. It is probable that fat body cells lack the enzyme(s) that cleaves peptides at dibasic sites. The discovery of BIGFLP suggests that the mature form of bombyxin depends on the type of cells where the bombyxin gene is expressed.

A remarkable feature of most bombyxin genes is a lack of any introns (Iwami, 2000), since vertebrate insulins consistently have two introns at conserved sites, one in the 5′-UTR and the other in the C-domain (Steiner et al., 1985). However, three of the recently identified bombyxin genes were found to have introns. *Bombyxin-V1* and *-V2* each had one intervening intron of different length in the 5′-UTR, and *bombyxin-Z1* contained two introns in the 5′ UTR and one intron within the C-domain (Aslam et al., 2011).

In the *Bombyx* genome, most bombyxin genes are clustered in two segments on chromosome 11 (25 genes) and an unidentified chromosome (6 genes), while forming gene pairs, gene triplets, or single genes. Other genes are located singly on chromosome 1 (3 genes), chromosome 9 (2 genes), and chromosome 11 (1 gene) (Iwami, 2000; Aslam et al., 2011).

## Bombyxin biosynthesis and release

### Bombyxin-producing cells

**Figure 1:**
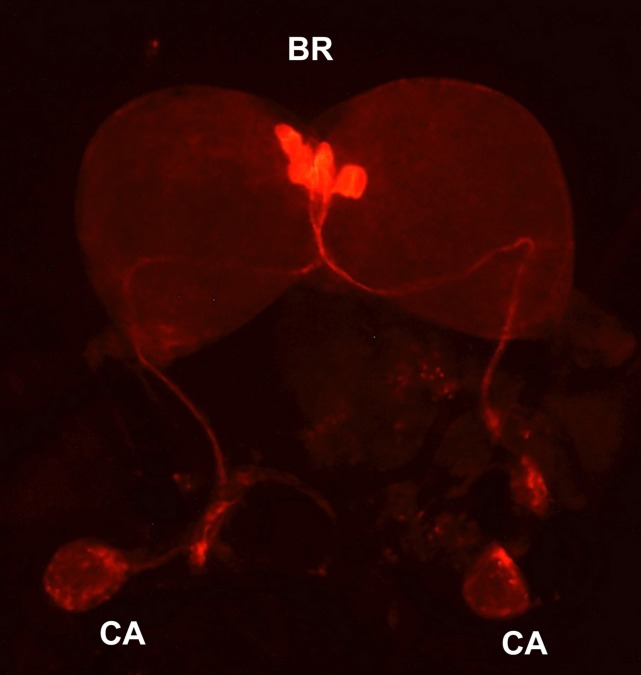
**Bombyxin-producing neurosecretory cells**. Whole-mount immunohistochemistry with anti-bombyxin-II mouse monoclonal antibody (A7B11) was performed on the brain-retrocerebral complex of the day-3 fifth instar larva of *B. mori*. BR, brain; CA, corpus allatum.

Bombyxin-producing cells were first identified by immunocytochemistry using a mouse monoclonal antibody (AN-I) generated against a synthetic decapeptide corresponding to the N-terminal portion of the A-chain of bombyxin-I (Mizoguchi et al., 1987). This antibody immunostained four pairs of large mid-dorsal neurosecretory cells of the brain and thick and dense nerve fibers around the periphery of the CA, suggesting that bombyxin is produced by these neurosecretory cells, axonally transported to and released from the CA. The axonal pathway of the bombyxin-containing neurons was not clear in this study, because immunohistochemistry was performed on brain sections. However, the subsequent study employing intracellular injection of Lucifer yellow (Ichikawa, 1991) or whole-mount immunohistochemistry (Figure 1) confirmed the connection between the medial neurosecretory cells (MNCs) in the brain and the axon terminals in the CA. These studies revealed that the axons originating from the four MNCs are fasciculated, traverse the midline of the brain to enter the contralateral lobe, and proceed along the ventral surface of the brain toward the retrocerebral nerve. The axons descend down the nerve, pass through the CC, and terminate in the CA with branches. Lucifer yellow injection in bombyxin-producing cells also revealed thick ipsilateral and thin contralateral dendrites as well as an abundance of varicosities along the branched axons over the periphery of the CA (Ichikawa, 1991).

Developmentally, the number of bombyxin-producing neurosecretory cells in the brain is consistently four throughout postembryonic development from hatching through adult eclosion (Mizoguchi et al., 1990). However, the immunoreactivity of the cells has been shown to markedly change during *Bombyx* development, especially in the fifth instar. For the first three days of the instar, the four pairs of cells are heavily stained but thereafter the stainability is impaired. Interestingly, two of these cells in each brain hemisphere are relatively intensely stained whereas the other two cells stained poorly. This unequal stainability of the cells might represent a pulsatile secretion of bombyxin. The immunoreactivity becomes weakest on the day of wandering and is then gradually regained (Mizoguchi et al., 1990).

The bombyxin-producing cells in the brain have also been identified by *in situ* hybridization. The bombyxin genes of families A–F, V, W, and Y are all expressed in four pairs of MNCs of the fifth larval brain (Iwami, 2000; Aslam et al., 2011), indicating that these cells are the exclusive source of bombyxin in the brain.

Early investigations by Northern hybridization could not detect bombyxin expression in tissues other than brain (Adachi et al., 1989; Iwami et al., 1990). However, RT-PCR analyses have disclosed that bombyxin genes are ubiquitously expressed in larval tissues, although the level of expression is very low if compared to that in the brain (Iwami et al., 1996). Moreover, it has recently been shown that some “bombyxin” genes are expressed predominantly in the fat body and/or gonads in a stage specific manner. Such genes include bombxin-X1, Y1 and ZI genes (Aslam et al., 2011). However, calling them “bombyxin” genes is controversial, because one of the products of these genes has been shown to be more like IGF rather than insulin in its structural features and physiological roles [see section *Bombyx* IGF-Like Peptide (BIGFLP)]. If bombyxin is defined as a structurally and functionally insulin-like peptide of *B. mori*, the IGF-like peptide should be called by other name. In this review, we discriminate the insulin-related peptides produced mainly by peripheral tissues from bombyxins, which are produced predominantly by the brain neurosecretory cells.

### Measurement of bombyxin

Bombyxin titers in the hemolymph have been determined by RIA (Saegusa et al., 1992; Masumura et al., 2000) or time-resolved fluoroimmunoassay (TR-FIA) (Satake et al., 1997). RIA was developed using a mouse monoclonal antibody (M7H2) generated against pure native bombyxin-II and ^125^I-labeled native bombyxin-II. The bombyxin concentration determined by this RIA was expressed by *Samia* units/ml, because a partially purified bombyxin was used as the standard, due to a paucity of the pure hormone. The lower limit of detection was equivalent to 0.2 *Samia* units (as determined by the *Samia* pupal assay) of bombyxin contained in the partially purified bombyxin. One *Samia* unit corresponds to 0.1 ng of bombyxin-I and -III and 0.4 ng of bombyxin-II (Nagasawa et al., 1984b). Therefore, the sensitivity of this RIA can be roughly estimated to be several tens of picograms, assuming that the anti-bombyxin-II antibody binds to any bombyxin species with the same affinity. In fact, however, the cross-reactivity of this antibody to other bombyxin species is not known. Nevertheless, this RIA provided a valuable opportunity to assess the physiological function of bombyxin and to analyze the regulation of its secretion. The titers of bombyxin immunoreactivity in the hemolymph were relatively constant during larval development with no correlation with ecdysteroid peaks, confirming that this hormone is not involved in the regulation of PG activity (Saegusa et al., 1992). Although the hemolymph titers of bombyxin immunoreactivity determined by RIA were remarkably high during pupa-adult development, this immunoreactivity in the pupal hemolymph was later proved not to represent bombyxin but to denote other bombyxin-related peptide. However, without this information about very high bombyxin titers in the pupal hemolymph, the recent discovery of an IGF-like peptide may not have been achieved [see section *Bombyx* IGF-Like Peptide (BIGFLP)]. The same RIA was applied to investigate the effects of starvation and feeding of larvae on bombyxin secretion (Masumura et al., 2000), revealing an important aspect of bombyxin secretion (see section Regulation of Bombyxin Release).

More recently, a TR-FIA based on a sandwich protocol has been developed using two kinds of monoclonal antibodies against synthetic bombyxin-II (A3A1 and A7B11), one of which was biotin-labeled and used as a tracer antibody, and the other used as a capture antibody (Satake et al., 1997). The lower limit of detection by this assay was 3 pg (bombyxin-II equivalents), much lower than that of the RIA. This TR-FIA was applied to measure bombyxin titers in the hemolymph of *Bombyx* adults, revealing that bombyxin is released immediately after eclosion in a sex-specific manner (Satake et al., 1999). The peak bombyxin titer was three times higher in males. However, the physiological relevance of the bombyxin release at this stage and its sex difference remains to be elucidated.

### Regulation of bombyxin release

The effects of starvation and refeeding on bombyxin secretion were investigated in *Bombyx* larvae. The release of bombyxin was monitored by measuring hemolymph titers and brain contents of bombyxin by RIA (Masumura et al., 2000). Following starvation for 3–6 h, the hemolymph titer decreased, while the brain content increased significantly. By contrast, refeeding after 6 h-starvation resulted in a quick recovery of the hemolymph bombyxin titer as well as a quick decrease in the brain bombyxin content. These results clearly demonstrated that bombyxin release is closely associated with feeding. In humans, insulin secretion is stimulated by hyperglycemia after a meal. The glucose levels in *Bombyx* hemolymph also change dramatically after starvation and refeeding. Therefore, the effect of glucose on bombyxin release was examined by injecting d-glucose into starved larvae. The brain bombyxin content decreased within 1 h after glucose injection in a dose-dependent manner. This result indicates that glucose serves as a common nutritional signal for inducing the release of both mammalian and insect insulins as a messenger for the “fed” state of the body.

It is unclear whether the bombyxin-producing cells directly respond to circulating glucose or not. In *D. melanogaster*, the secretion of insulin-like peptides (DILPs) from the brain also depends on the nutritional conditions, but the availability of nutrients is remotely sensed by the fat body, which in turn regulates DILP secretion through humoral factors (Géminard et al., 2009; Bai et al., 2012; Rajan and Perrimon, 2012). In this case, the major nutritional signal in the diet is not glucose but amino acids (Géminard et al., 2009).

## Bombyxin actions

### Regulation of trehalose metabolism

When bombyxin was purified from *B. mori*, no physiological action of this peptide was known in *B. mori* from which it was derived (see section Discovery of Bombyxin). Prior to the identification of bombyxin as an insulin-related peptide, however, the presence in several insects of insulin-like hypoglycemic hormones had been repeatedly suggested (see section Historical Background). Thus, soon after a synthetic bombyxin-II was obtained (Nagata et al., 1992), the effects of bombyxin on sugar metabolism were investigated. Bombyxin-II lowered the concentration of the major hemolymph sugar, trehalose, in a dose-dependent manner when injected into neck-ligated *Bombyx* larvae (Satake et al., 1997).

Bombyxin-II injection also led to the elevation of trehalase activity in the midgut and muscle (Satake et al., 1997). Trehalase located on the cell surface is assumed to hydrolyze trehalose in the hemolymph to glucose for transport into the cells (Shimada and Yamashita, 1979; Azuma and Yamashita, 1985). Therefore, these observations suggest that bombyxin induces hypotrehalosemia by promoting the hydrolysis of hemolymph trehalose to glucose and thereby facilitating its incorporation into tissues. However, such effects of bombyxin on trehalose metabolism may be larval stage-specific, because bombyxin-II injection into adults did not result in hypotrehalosemia (Satake et al., 1999).

Glucose also constitutes the hemolymph sugar in *B. mori*, although its concentration is very low as compared with that of trehalose. Interestingly, bombyxin-II injection did not affect the glucose concentration in the hemolymph (Satake et al., 1997).

### Regulation of glycogen metabolism

In mammals, insulin not only promotes glucose uptake by adipose tissue and muscle but also facilitates glycogen production in some tissues including liver and muscle (Newsholme et al., 1992; Saltiel and Kahn, 2001). Therefore, the effect of bombyxin on tissue glycogen content was also examined. Unexpectedly, glycogen content in the fat body, a major glycogen storage tissue, was significantly decreased, but not increased, after bombyxin-II injection (Satake et al., 1997).

The effect of bombyxin on the activity of glycogen phosphorylase, a key enzyme of glycogenolysis, was also examined. When 10 ng of bombyxin-II was injected, the percentage of active glycogen phosphorylase was significantly increased, providing a biochemical basis for the glycogen-reducing effect of bombyxin (Satake et al., 1997).

It is worth noting that hemolymph trehalose and fat body glycogen are both major storage forms of carbohydrates in insect. Thus, the results of these investigations suggest that bombyxin likely functions to facilitate the use of energy reserves.

### Regulation of tissue growth

The growth-promoting action of bombyxin on wing imaginal discs has been demonstrated in the butterfly *Precis coenia* (Nijhout and Grunert, 2002). Although the wing disc of the final instar larva does not grow *in vitro* in the standard tissue culture medium without supplements, it does grow in the medium supplemented with an optimal concentration of 20-hydroxyecdysone (20E, 0.1 ng/ml) and with hemolymph taken from growing larvae. Assuming that the factor in the hemolymph responsible for the promoted growth of the disc is bombyxin, the effect of bombyxin-II on the disc growth *in vitro* was examined, using mitotic rate as an indicator of its growth. Bombyxin-II elevated the mitotic rate in a dose-dependent manner when added together with 0.1 mg/ml 20E to the culture. Moreover, the growth-promoting effect of the hemolymph was abolished when the hemolymph was pretreated with bombyxin antibodies (AN-I and guinea pig polyclonal antibody against synthetic bombyxin-II)-bound column to remove bombyxin, leading the authors to conclude that bombyxin is a growth factor for wing imaginal discs (Nijhout and Grunert, 2002). A similar effect of bombyxin-II on wing disc growth has also been reported in *M. sexta* (Nijhout et al., 2007).

Bombyxin-II also promotes cell proliferation in the hematopoietic organ (HPO) of *B. mori*. Cell proliferation in the larval HPOs cultured *in vitro* is markedly promoted by *B. mori* larval hemolymph. Thus, the active factor in the hemolymph has again been presumed to be bombyxin. When the HPOs from day-1 fifth instar larvae were cultured for 48 h with bombyxin-II, the number of discharged hemocytes increased in a dose-dependent manner (Nakahara et al., 2006). Because the hemocyte discharge is linked to cell proliferation (Nakahara et al., 2003), this result shows mitogenic activity for bombyxin-II. In contrast to the above study with the *Precis* wing discs, bombyxin-II was still effective in the absence of 20E. 20E was also mitogenic to the HPO irrespective of the presence of bombyxin-II. Despite the clear action of bombyxin-II on the HPO, the authors concluded that bombyxin is not the primary effector in the larval hemolymph, because anti-bombyxin-II antibodies (a mixture of M7H2, A3A1, A7B11, and guinea pig polyclonal antibody) were unable to neutralize the mitogenic activity of the hemolymph. However, this conclusion might be premature, because bombyxin is highly heterogeneous and the anti-bombyxin-II antibodies are only able to recognize a part of many bombyxin molecular species(Saegusa et al., 1992).

The effect of bombyxin on the systemic growth of lepidopteran insects including *B. mori* has not been investigated due to the difficulties in applying genetic approaches to analyze bombyxin actions in this order of insects (Terenius et al., 2011). However, such an effect of bombyxin homologues has been extensively studied in *D. melanogaster*, which has eight insulin-like peptides (DILP1-8) (Brogiolo et al., 2001; Colombani et al., 2012; Garelli et al., 2012). Among them, *dilp2, −3, and −5* are mainly expressed in the MNCs of the brain as with bombyxin (Brogiolo et al., 2001; Ikeya et al., 2002; Rulifson et al., 2002; Broughton et al., 2005; Nässel, 2012) and their products are thus thought to be functional homologues of bombyxins. When individual *dilps* (*dilp1-7*) were overexpressed, a proportionate increase in body size was observed in adult flies (Brogiolo et al., 2001; Ikeya et al., 2002), with *dilp2* showing the highest potency for growth promotion (Ikeya et al., 2002). The *dilp2-*overexpressed flies exhibited larger wing size due to increases in both cell size and cell number. In contrast, when the DILP-producing MNCs in the brain were genetically ablated using a *dilp2* promoter to express the apoptotic gene, *reaper*, larval growth was severely retarded, puparium formation delayed, and adult size reduced. All these phenotypes were rescued by ubiquitously expressing *dilp2* using heat-shock promoter (Rulifson et al., 2002). These results clearly demonstrate that the DILPs secreted from the MNCs are potent regulators of the systemic growth of *Drosophila*.

### Other known actions

A highly purified preparation of bombyxin induced meiosis in the ovaries that were taken from young *Bombyx* larvae and cultured *in vitro* (Orikasa et al., 1993). However, the authors proposed that bombyxin merely stimulates the ovaries to produce ecdysteroids, which in turn induce meiosis of the ovarian cells, because 20E also induces meiosis at a very low concentration (Orikasa et al., 1993) and because the ovaries from the fourth instar *Bombyx* larvae secrete ecdysteroids *in vitro* (Orikasa, unpublished).

It has also been reported that synthetic bombyxin-II induces a series of morphological changes in BM-N4 cells (Tanaka et al., 1995), a cell line established from ovarian tissues of *B. mori* (Volkman and Goldsmith, 1982). After bombyxin administration, most of the cells became bigger and round in shape and aggregated to form clumps. Some other cells were tightly attached to the bottom of the culture flask forming a spindle-like or fibroblastic shape (Tanaka et al., 1995). These morphological changes were induced by bombyxin at a concentration of 1 nM or higher. Five other *Bombyx* cell lines, all of which were derived from embryos, did not respond to bombyxin at concentrations up to 1 μ M.

A bombyxin-like peptide of *M. sexta* has been suggested to be involved in the pupal commitment of wing imaginal discs (Koyama et al., 2008). At the beginning of the final larval instar, wing discs are committed to initiate larval-pupal development. Juvenile hormone (JH) prevents this commitment in earlier instars and in starved final instar larvae, but nutrient intake overcomes this effect of JH in the latter (Truman et al., 2006). Thus, an insulin/IGF signal was hypothesized to mediate the effect of nutrients. When wing discs from freshly molted final instar larvae were cultured *in vitro* with methoprene, a JH mimic, the expression level of *broad*, a molecular marker for pupal commitment, was suppressed. The suppression effect of JH, however, was overcome by the administration of partially purified *Manduca* bombyxin or bovine insulin, supporting the above-mentioned hypothesis.

As described in section Discovery of Bombyxin, bombyxin did not stimulate *Bombyx* PGs in the debrained pupal assay or in the conventional *in vitro* PG assay. Recently, however, it was demonstrated that bovine insulin stimulates ecdysteroidogenesis in *Bombyx* PGs during a long-term incubation period (Gu et al., 2009). A significant increase in ecdysteroid secretion by the PGs was observed after 8 h of incubation with insulin at a concentration of 1.7 μ M or higher. In addition, insulin also stimulated DNA synthesis and cell viability of PGs, as assayed after 48 h of incubation. The PG-stimulating action of insulin was also verified by *in vivo* experiments: injection of insulin into day 6 last instar larvae significantly increased both the hemolymph ecdysteroid titers and *in vitro* ecdysteroidogenic activity of the PGs 24 h after the injection. Although bovine insulin was used in this study, the results strongly suggest that bombyxin, intrinsic insulin of *B. mori*, has the same long-term actions on the PGs. The effects of insulin-like peptides on ecdysteroidogenesis in PGs are also demonstrated in *D. melanogaster* (Caldwell et al., 2005; Colombani et al., 2005; Mirth et al., 2005; Walkiewicz and Stern, 2009).

## *Bombyx* IGF-like peptide (BIGFLP)

### Discovery of BIGFLP

As described in section Measurement of Bombyxin, the hemolymph bombyxin titer, as measured by RIA, is remarkably high during metamorphosis (Saegusa et al., 1992). The titers were so high the authors suspected that the immunoreactivity in the pupal hemolymph did not represent bombyxin, because it was unlikely that only eight neurosecretory cells in the brain could secrete such large amounts of bombyxin. Therefore, pupal hemolymph was analyzed by Western blotting using M7H2 antibody, the same antibody as used for RIA, to estimate the molecular mass of the immunoreactive material. It was 8 kDa, slightly larger than that of bombyxin-II and similar to that of a bombyxin precursor polypeptide. Thus, it was decided to purify this peptide to determine its structure. The peptide was effectively enriched from pupal hemolymph using an M7H2 antibody-bound affinity column and further purified to homogeneity through only two steps of HPLC (Okamoto et al., 2009a). The amino acid sequence of the peptide was determined by a combination of N-terminal sequencing, *Bombyx* genome search and MALDI TOF-MS analysis. The peptide had high homology with bombyxins and most interestingly it contained the C-domain within the molecule. Although the C-domain was flanked by dibasic amino acid motifs, it was obvious that this peptide is secreted into hemolymph as a single chain peptide like IGFs, because it was purified from the hemolymph. For this and some other reasons described below, this peptide was named BIGFLP (Okamoto et al., 2009a).

### BIGFLP-producing cells

Although all known bombyxins are mainly produced by MNCs in the brain (Mizoguchi et al., 1987; Iwami, 2000), expression analysis by real-time quantitative RT-PCR (qRT-PCR) revealed that BIGFLP is predominantly produced by the fat body (Okamoto et al., 2009a). The fat body is the functional equivalent of the vertebrate liver, which is the major source of circulating IGFs. BIGFLP gene expression in the fat body was very low during larval stages but dramatically increased during metamorphosis. Production of BIGFLP by the fat body was also confirmed by immunohistochemistry with a BIGFLP-specific monoclonal antibody (D7H3) generated against its C-domain (Okamoto et al., 2009a, 2011). BIGFLP-producing tissues and stages were systematically surveyed by means of immunohistochemistry, *in situ* hybridization, as well as qRT-PCR (Okamoto et al., 2011). These analyses revealed that BIGFLP is produced not only by the fat body but also by the brain and gonads in a stage-specific manner (Okamoto et al., 2011). The BIGFLP-producing cells in the brain were identical to the cells that produce bombyxins, but the temporal expression pattern of BIGFLP was clearly different from that of bombyxins. BIGFLP is produced in the brain only after the penultimate (fourth) instar stage. In the ovary, BIGFLP expression was observed in the ovariole sheath, which wraps around an array of follicles, during the wandering and early pupal stages. The sheath of testis also produces BIGFLP during pupa-adult development.

### Regulation of BIGFLP production and release

The temporal expression patterns of the BIGFLP gene in the fat body and gonads suggested that BIGFLP production in these organs is regulated by ecdysteroids, which are released concurrently. This hypothesis was verified though the experiments where these tissues were cultured *in vitro* with 20E (Okamoto et al., 2009a, 2011). In contrast, *BIGFLP* expression in the brain was not induced by 20E (Okamoto et al., 2011). These results indicate that the regulatory mechanisms of BIGFLP gene expression differ across tissues.

BIGFLP titers in the hemolymph were determined by TR-FIA using two antibodies, one of which (capture antibody) was a BIGFLP-specific mouse monoclonal antibody (D11E12) against its C-domain and the other (tracer antibody) was anti-bombyxin-II guinea pig polyclonal antibody that cross-reacts with BIGFLP. The BIGFLP titers were remarkably high, with the titer rising early in the pupal stage to 800 nM in females and 200 nM in males. These values are much more similar to those of IGFs in human adults (20–80 nM) (Humbel, 1990) than to those of bombyxin and insulin, which are of the order of 100 pM (Andersen et al., 1993; Satake et al., 1999).

### BIGFLP actions

Lepidopteran pupae initiate adult development shortly after pupal ecdysis, when larval tissues degenerate, while adult tissues including reproductive system, imaginal discs and flight muscles undergo growth and differentiation. The temporal secretion pattern of BIGFLP suggested that BIGFLP regulates growth and development of adult tissues during metamorphosis. The potential growth-promoting effect of BIGFLP was investigated using an *in vitro* tissue culture system. When genital imaginal discs were cultured with BIGFLP, the tissue size, protein content and cell number of the discs were significantly increased after 5 days. BIGFLP also promoted BrdU incorporation not only in genital discs but also in sperm ducts, flight muscle anlagen and wing discs. Interestingly, no or little promotion of BrdU incorporation by BIGFLP was observed in the fat body, midgut and epidermis, all of which are degenerated or reconstructed during adult development, suggesting that BIGFLP functions as a growth hormone to regulate adult tissue development in *B. mori* (Okamoto et al., 2009a).

Although the dominant source of BIGFLP in the hemolymph is the fat body, several other tissues including brain and gonads also produce BIGFLP (see section BIGFLP-Producing Cells). For example, the ovariole sheath produces BIGFLP during the period when the ovaries undergo rapid growth and development. BIGFLP expression is especially high in the area where follicles are immature. Interestingly, the *Bombyx* insulin-like receptor gene is also predominantly expressed in the early vitellogenic ovaries (Swevers and Iatrou, 2003). Thus, ovariole sheath-derived BIGFLP may regulate the early follicular growth in a paracrine manner. Thus, BIGFLP may exert its effects through both endocrine and paracrine pathways, as do the vertebrate IGFs.

## IGF-like peptide in *drosophila*

### Candidates for IGF-like peptides in other insects

**Figure 2:**
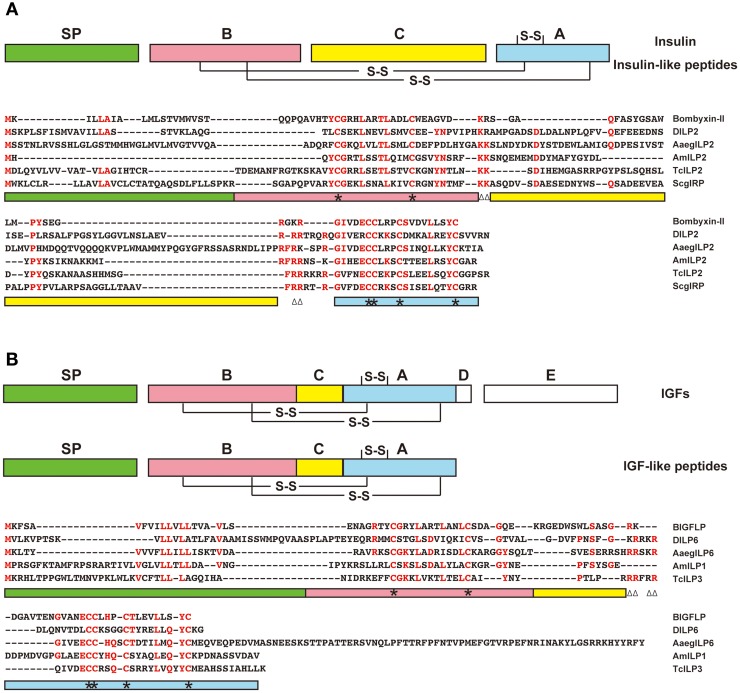
**Predicted insulin-like and IGF-like peptides in insects**. Amino acid sequences of the representatives of putative insulin-like **(A)** and IGF-like **(B)** peptides from *B. mori* (bombyxin-II and BIGFLP), *D. melanogaster* (DILP2 and 6), *A. aegypti* (AaegILP2 and 6), *A. mellifera* (AmILP2 and 1), *T. castaneum* (TcILP2 and 3), and *Schistocerca gregaria* (ScgIRP) are aligned. Highly conserved amino acid residues are shown in red. Color bars below the alignment indicate the predicted domains in the precursor peptides: green, signal peptide; red, B-domain; yellow, C-domain; blue, A-domain. Asterisks denote cysteine residues, and paired triangles denote potential cleavage sites (dibasic amino acids).

Are IGF-like peptides present in insects other than *B. mori*? Based on sequence homology, no obvious orthologs of BIGFLP have been identified in other insects, because the amino acid sequences of insect insulin-related peptides are highly diverged between insect orders, except for some critical residues necessary for tertiary structure formation. One of the characteristic features of IGFs as compared to proinsulin is a shortened C-domain. In this regard, some insulin-related peptides in insects are more similar to IGFs than to insulin (Figure 2). Such peptides are found in *D. melanogaster* (DILP6) (Brogiolo et al., 2001), *A. mellifera* (AmILP1) (Wheeler et al., 2006), the red flour beetle *Tribolium castaneum* (TcILP3) (Li et al., 2008), and the mosquito *Aedes aegypti* (AaegILP6) (Riehle et al., 2006).

Another characteristic feature of the IGF-like peptide in *B. mori* is a very high level of gene expression in the fat body during metamorphosis. Therefore, insulin-related genes in other insects showing such an expression pattern are good candidates for being IGF-like peptides. Although bombyxin genes and a majority of known insect insulin-related genes are mainly expressed in MNCs of the brain (Mizoguchi et al., 1987; Iwami, 2000; Brogiolo et al., 2001; Ikeya et al., 2002; Rulifson et al., 2002; Broughton et al., 2005; Nässel, 2012), some insulin-related genes are expressed outside the brain (Brogiolo et al., 2001; Riehle et al., 2006).

### DILP6 as a *drosophila* IGF-like peptide

In *D. melanogaster*, developmental changes in the gene expression of seven DILPs were investigated from embryo to adult (Okamoto et al., 2009b; Slaidina et al., 2009). These analyses revealed that only *dilp6* was expressed predominantly during the late third instar and during pupa-adult development, at remarkably high levels. Furthermore, *dilp6* exhibited the highest expression in the fat body. These characteristics of *dilp6* expression, together with the structural feature of DILP6, led to its identification as a *Drosophila* IGF-like peptide. Interestingly, *dilp6* expression in the fat body is induced *in vitro* by 20E (Okamoto et al., 2009b; Slaidina et al., 2009), as is *BIGFLP* (Okamoto et al., 2009a). The ecdysteroid-induced expression of *dilp6* in the fat body is not affected by cycloheximide, a protein synthesis inhibitor, suggesting that *dilp6* expression is directly induced by 20E (Okamoto et al., 2009b). The expression of *dilp6* in the fat body is also up-regulated by starvation of larvae through direct induction by Forkhead box O (FOXO) transcription factor, independent of the ecdysteroids (Slaidina et al., 2009), suggesting that DILP6 also plays some roles during starved conditions. A recent study has shown that *dilp6* expression in the adult fat body is also regulated by FOXO (Bai et al., 2012). This FOXO-inducible *dilp6* expression is suggested to be involved in the compensatory regulation between fat body-derived and brain-derived DILPs (Grönke et al., 2010; Bai et al., 2012).

### DILP6 functions

Several research groups independently generated *dilp6* mutants and revealed that these mutants result in ~10% reduced final adult body size and weight (Okamoto et al., 2009b; Slaidina et al., 2009; Zhang et al., 2009; Grönke et al., 2010). The hair density analysis demonstrated that *dilp6* mutants have normal cell size but reduced cell number, which likely accounts for the reduction in body size (Okamoto et al., 2009b). Based on the temporal *dilp6* expression pattern, DILP6 was expected to regulate growth during metamorphosis. The developmental changes in body weight were investigated in the *dilp6* mutants from the beginning of the 3rd instar to adult emergence (Okamoto et al., 2009b). During the feeding period in the 3rd instar, the growth rate was the same as controls. However, the difference in body weight between controls and *dilp6* mutants was manifested during the post-feeding period. The *dilp6* mutants lost weight at a higher rate and emerged as smaller adult flies. During the wandering and adult developmental stages, when animals never feed, the nutrient supply is limited to the stored nutrients that have been accumulated during the feeding period. Therefore, this *dilp6* mutant phenotype suggests a role for DILP6 in regulating the utilization efficiency of nutrient stores during the post-feeding stages. This phenotype is rescued by fat body-specific expression of *dilp6* during the post-feeding period (Okamoto et al., 2009b). Furthermore, when *dilp6* was overexpressed during the post-feeding period, the body weight of adult flies was increased compared with control animals, showing that the effect of DILP6 on adult size is closely related to its expression level (Okamoto et al., 2009b). Similar results were also obtained by using *Gal4/Gal80^ts^* temporal conditional expression system to induce *dilp6* RNAi or overexpression during the post-feeding period (Slaidina et al., 2009). These studies demonstrate that DILP6 is involved in the regulation of postfeeding growth (Okamoto et al., 2009b; Slaidina et al., 2009).

Recent several studies have demonstrated that DILP6 act locally within the central nervous system (Chell and Brand, 2010; Sousa-Nunes et al., 2011; Avet-Rochex et al., 2012). A subset of glia expresses *dilp6* during the feeding stages of larvae, and its expression is inhibited by starvation. This nutrient-dependent expression and/or secretion of DILP6 activate the insulin/IGF signaling in the adjacent neuroblasts, thereby leading to their exit from quiescence (Chell and Brand, 2010; Sousa-Nunes et al., 2011). Moreover, DILP6 has also been suggested to regulate the proliferation of perineural and cortex glia in the larval brain (Avet-Rochex et al., 2012). Thus, the fat body-derived DILP6 promotes systemic growth in an endocrine fashion, while the glia-derived DILP6 regulates neuroblast reactivation and gliogenesis in a paracrine/autocrine manner.

## Concluding remarks

**Figure 3:**
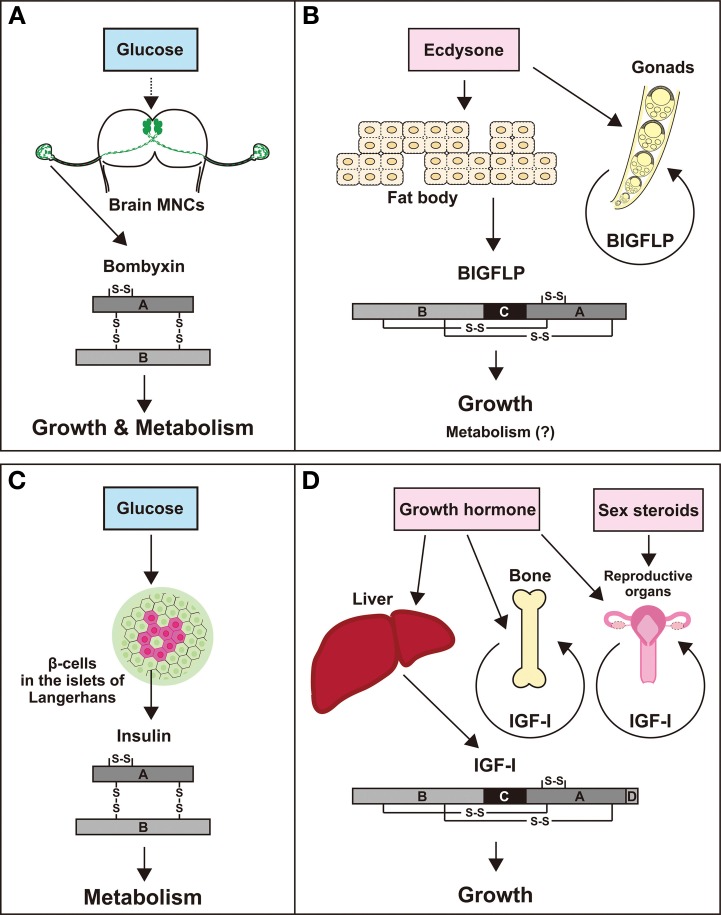
**Comparison of bombyxin and BIGFLP of *B. mori* with insulin and IGF-I of humans**. High similarities are noted between bombyxin **(A)** and insulin **(C)** and between BIGFLP **(B)** and IGF-I **(D)** in the structural feature, sites of production, secretion control, and actions.

Two classes of insulin-related peptides were discovered and characterized in *B. mori*; one is bombyxin, which is a functional counterpart of vertebrate insulin, and the other is BIGFLP, which is more similar to IGFs rather than to insulin in the structural and biological traits. However, these two peptides are not complete homologues of insulin and IGF, because they are presumed to share the same receptor (Okamoto et al., 2009b) and therefore have essentially the same actions, unlike insulin and IGF, each of which has its own receptor. Nevertheless, it is still conceivable that bombyxin and BIGFLP are functional counterparts of insulin and IGF, respectively, for the following reasons. (1) The essential role of insulin and bombyxin, both of which are secreted after food intake, is to convey information about the nutritional conditions of the body to the peripheral tissues. (2) IGF and BIGFLP are secreted to promote tissue growth under the control of specific hormones that are released at specific stages of development. (3) IGF and BIGFLP are mainly produced by homologous tissues, the liver and the fat body, respectively, and are also produced by other peripheral tissues. Thus, although the actions on target tissues may be the same, the physiological functions of the two hormones are distinct. It is likely that bombyxin serves as a link between food intake and larval growth, while BIGFLP plays a role in facilitating metamorphic growth under the control of the developmental signal, ecdysteroid. Both hormones may also regulate energy metabolism to support the growth of the animal. Figure 3 compares the corresponding insulin-family peptides between *B. mori* and humans.

Despite striking functional similarities between insulin and bombyxin and between IGF and BIGFLP, it is unlikely that the ancestral insulin and IGF had already existed even prior to the divergence of protostomes and deuterostomes, because BIGFLP is much more similar to bombyxin than IGFs in its amino acid sequence. Both classes of insulin-family peptides may have evolved independently in vertebrates and insects (even in different groups of insects) from a single ancestral insulin/IGF peptide, as discussed by Okamoto et al. (2009b). In insects, this ancestral peptide may have been produced by various tissues including the brain and fat body but differentially regulated at the level of gene expression and/or of secretion in a tissue specific manner. We surmise that in the course of evolution of each insect group, gene duplications followed by divergence may have led to the brain-specific expression of an insulin-like gene(s) and to the fat body-specific expression of an IGF-like gene(s).

### Conflict of interest statement

The authors declare that the research was conducted in the absence of any commercial or financial relationships that could be construed as a potential conflict of interest.
